# An Outer Membrane Protein YiaD Contributes to Adaptive Resistance of Meropenem in Acinetobacter baumannii

**DOI:** 10.1128/spectrum.00173-22

**Published:** 2022-04-04

**Authors:** Lei Han, Yiyuan Gao, Yuqing Liu, Siyu Yao, Shuyan Zhong, Sirui Zhang, Jingdan Wang, Peng Mi, Yurong Wen, Zhenlin Ouyang, Jing Zhang, Mona Mohamed Al-Shamiri, Pu Li, Shaoshan Han

**Affiliations:** a Department of Microbiology and Immunology, School of Basic Medical Sciences, Xi’an Jiaotong University Health Science Center, Xi’an, China; b School of Public Health, Xi'an Jiaotong University Health Science Center, Xi’an, China; c Department of Laboratory Medicine, Shaanxi Provincial People’s Hospital, Xi’an, China; d The First Affiliated Hospital of Xi'an Jiaotong University, Xi’an, China; e The Key Laboratory of Environment and Genes Related to Disease of Ministry of Education, Xi'an Jiaotong University Health Science Center, Xi’an, China; f Department of Laboratory Medicine, the Second Affiliated Hospital of Xi'an Jiaotong University, Xi'an, China; g Department of Hepatobiliary Surgery, the First Affiliated Hospital of Xi’an Jiaotong University, Xi'an, China; University of Maryland School of Pharmacy

**Keywords:** YiaD, *Acinetobacter baumannii*, meropenem, adaptive resistance, resistance evolution

## Abstract

Acinetobacter baumannii is an important nosocomial pathogen that can develop various resistance mechanisms to many antibiotics. However, little is known about how it evolves from an antibiotic sensitive to a resistant phenotype. In this study, we investigated the transition of outer membrane proteins (OMPs) under antibiotic stress and identified YiaD as an OMP marker involved in the development of adaptive resistance to meropenem (MEM) in A. baumannii. Following stimulation of a carbapenem-sensitive strain AB5116 with sub-MIC of MEM, *yiaD* showed significantly decreased expression, and this decrease continued with prolonged stimulation for 8 h. The downregulation of *yiaD* was not only observed in clinically sensitive strains but also in 45 carbapenem-resistant isolates that produced the β-lactamases TEM and OXA-23. However, the extent of the reduction of *yiaD* expression in resistant strains was less than that in sensitive strains. Lack of *yiaD* resulted in a 4-fold increase in the MIC of AB5116 to MEM. The same level of depressed susceptibility induced by *yiaD* deletion was observed in both a growth curve test and a survival rate assay. Moreover, the colony shape became enlarged and irregular after loss of *yiaD*, and the biofilm formation ability of A. baumannii was influenced by YiaD. These results suggest that YiaD could respond to the stimulus of MEM in A. baumannii with a downregulation trend that kept pace with the prolonged stimulation time, indicating that it participates in various routes to benefit MEM resistance evolution in both carbapenem-sensitive and -resistant A. baumannii strains.

**IMPORTANCE** Acinetobacter baumannii can develop various resistance mechanisms to carbapenems. However, the factors involved in the evolutionary process that leads from transition to the sensitive to resistant phenotype are not clear. The outer membrane protein YiaD of A. baumannii was downregulated under the stress of meropenem (MEM), and its expression level was continuously reduced with prolonged stimulation time. The downregulation of *yiaD* was not only observed in sensitive strains but also in carbapenem-resistant isolates producing the β-lactamases TEM and OXA-23. However, the extent of *yiaD* reduction was less in resistant strains than in sensitive strains. Lack of *yiaD* resulted in an increased MEM MIC, enlarged and irregular colonies, and decreased biofilm formation ability. These results suggest that YiaD responds to MEM stimulus in A. baumannii and participates in the adaptive resistance of MEM in both carbapenem-sensitive and -resistant strains.

## INTRODUCTION

Acinetobacter baumannii is an important nosocomial pathogen that can lead to a variety of infections, including ventilator-associated pneumonia, bloodstream infections, and meningitis (1). Moreover, A. baumannii has been reported to be associated with high mortality in intensive care units (ICUs), especially among critically ill patients (2). This is mainly because of its extraordinary ability to attach to abiotic surfaces and resist desiccation, as well as its multidrug and disinfectant resistance (3–5).

Antibiotic resistance is a remarkable property that helps A. baumannii survive in antibiotic-stressed environments, resulting in the organism posing a threat to humans and complicating treatments (6). Multidrug-resistant (MDR), extensively drug-resistant (XDR), and pan-drug-resistant (PDR) nosocomial isolates are now commonly found in hospitals (7) and community-dwellings, as well as in nursing homes, where they are closely related to adverse outcomes in elderly persons (8).

The most notorious drug-resistant strain is the carbapenem-resistant A. baumannii (CRAB). Indeed, this organism was listed as an “urgent threat” by the Centers for Disease Control and Prevention (CDC) in the 2019 Antibiotic Resistance Threats Report (6). Carbapenems are among the antimicrobials of last resort used for treatment of MDR infections, among which imipenem (IPM) and meropenem (MEM) are the most frequently used in clinics. However, their widespread use has led to increased resistance to these materials worldwide (9–11). In China, the average resistance rate of A. baumannii to IPM and MEM increased from 32.9% and 41.3% to 77.1% and 78.1%, respectively, from 2005 to 2018 (12). Moreover, a steady high resistance ratio toward these two antibiotics in elderly patients from 2014 to 2019 was reported by the China Antimicrobial Resistance Surveillance System (CARSS), with ratios of 56.7% to 61.0% being reported for IPM and 58.0% to 61.4% for MEM (13). These CRAB isolates are often resistant to multiple antibiotics (14), making them a great challenge in infection control.

Antibiotic resistance of bacteria can be intrinsic, acquired, or adaptive (15, 16). Intrinsic resistance refers to the resistance exhibited due to the inherent properties of a bacterium (17). As a Gram-negative bacterium, A. baumannii has an outer membrane that serves as a natural barrier of permeability. This membrane contains many components reportedly related to carbapenem resistance, including porin (OmpA, CarO, and OprD), capsular polysaccharides, and lipopolysaccharides (18). Efflux pumps are also associated with intrinsic antibiotic resistance (19). However, A. baumannii is well known to have a high capacity for acquired resistance because it has a tremendous genetic plasticity that results in a high capacity to acquire antimicrobial resistance traits, such as β-lactamases (7). Many of these enzymes are associated with transferable elements such as insertion sequences, integrons, and plasmids, which play major roles in carbapenem-resistance (19). Although investigations of resistant strain have revealed these mechanisms, less is known about how A. baumannii are transformed from a drug-sensitive to a drug-resistant phenotype. Adaptive resistance is defined as the resistance to antibiotics induced by specific signals, such as stress or sub-inhibitory levels of antibiotics (17). Adaptive resistance is transient and driven by differential expression of various genes, including those encoding efflux pumps and porins, which allows the bacteria to respond rapidly to antibiotic challenges and external stresses (15, 17, 20). Despite its importance to the development of resistance, factors involved in adaptive resistance in A. baumannii have not been thoroughly investigated.

In this study, we report an outer membrane protein (OMP) YiaD, which may play a role in the MEM adaptive resistance in A. baumannii.

## RESULTS

### RNA-Seq analysis.

To identify potential adaptational genes of A. baumannii involved in the stress response of MEM, RNA-Seq was conducted to demonstrate the transcriptional profile transition at a sub-MIC (0.5 × MIC, 0.0625 μg/mL) for 30 min.

**FIG 1:**
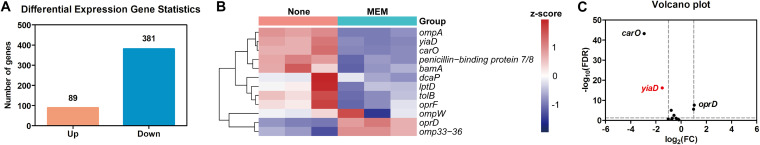
Analysis of RNA-Seq results and changes in the expression of outer membrane proteins (OMPs). (A) Statistics of differential expressed genes in AB5116 after treatment with 0.5 × MIC MEM. (B) Expressional heatmap of 12 OMP genes in three replicate RNA-Seq samples. The fragments per kilobase of transcript per million mapped reads (FPKM) values were standardized based on the Z-score using the formula Z = (x − μ)/σ. (C) Volcano plot of gene expression changes after treatment with MEM. Differentially expressed genes (DEGs) were selected using the standard of |log_2_(FC)| ≥ 1 and FDR < 0.05.

A total of 470 genes were found to be differentially expressed under the stress of MEM, among which 89 were upregulated and 381 were downregulated (Fig. 1A). Carbapenem resistance in A. baumannii is mainly mediated by alterations in OMPs, overexpression of efflux pumps, changes in penicillin-binding proteins, and production of β-lactamases (9). In our RNA-Seq analysis, efflux pump expression was primarily decreased, there was no significant difference between the majority of them. This performance is in conflict the function of the efflux pump, as one would expect the efflux pump expression to increase, thus increasing bacterial resistance to antibiotics. Additionally, no change in the expression of penicillin-binding proteins nor in the production of β-lactamases was found in AB5116. Several OMPs, including OmpA, CarO, and OprD are reportedly crucial to drug resistance; thus, we investigated the expression of 11 frequently reported OMPs in A. baumannii, OmpA, CarO, OprD, Omp 33–36 kDa, TolB, DcaP, BamA, OprF, LptD, OmpW, and penicillin-binding protein 7/8 (1, 21–23), as well as an OmpA-like protein, YiaD (24). Two of these showed an upward transcription trend, while 10 showed a downward transcription trend when the strain was under the stress of MEM (Fig. 1B). However, only *carO*, *yiaD*, and *oprD* could be regarded as differentially expressed genes (DEGs) (Fig. 1C), among which *carO* and *yiaD* expression was downregulated and *oprD* expression was upregulated. Previous studies demonstrated that CarO and OprD were linked with carbapenem resistance (21); however, this study is the first to demonstrate that YiaD exhibits distinct transcription that is associated with MEM adaptation and resistance evolution, although its function remains elusive.

### *yiaD* was downregulated under MEM pressure.

**FIG 2:**
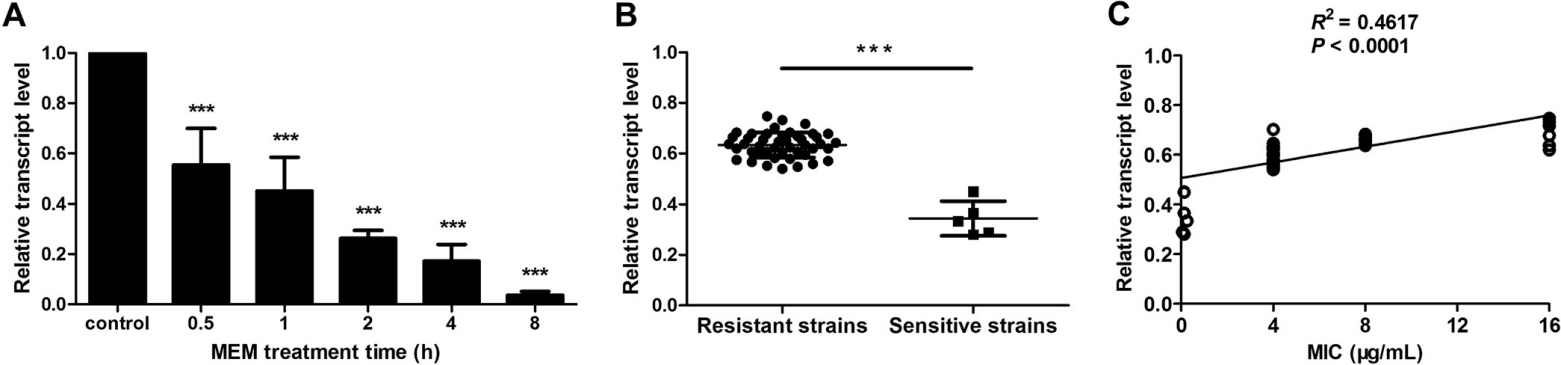
Time-dependent and strain-dependent features of *yiaD* expression under MEM stress. (A) Changes in *yiaD* expression after exposure of AB5116 to MEM for different times. (B) Decreased expression of *yiaD* in clinical strains under MEM stress for 2 h. ***, *P < *0.001. (C) Correlation between MIC value and decreased *yiaD* expression level in clinical isolates under MEM stress.

To verify the RNA-Seq data, RT-qPCR was used to confirm the decreased expression of *yiaD* under the stress of MEM. Exposure of AB5116 to sub-MIC levels of MEM for 30 min resulted in a great decrease in the expression level of *yiaD* (Fig. 2A, 0.5 h). Because MEM is generally administered every 8 h in clinics to maintain the blood concentration, bacteria are continually exposed to an antibiotic environment during this time; therefore, the observation period was prolonged to 8 h. As shown in Fig. 2A, the transcription of *yiaD* decreased remarkably in a time-dependent manner. Specifically, the relative transcript levels of the samples stimulated by MEM decreased to 0.555, 0.452, 0.263, 0.172, and 0.037 at 0.5 h, 1 h, 2 h, 4 h, and 8 h, respectively, relative to the control.

Because an obvious decline of *yiaD* expression was observed in AB5116 after treatment with MEM for 2 h, more clinical strains were tested at this time point to verify the results. Among the 50 collected isolates, 45 were carbapenem-resistant and five were carbapenem-sensitive. As shown in Table 1, β-lactamases were produced in carbapenem-resistant strains but not in sensitive strains. Although the OXA-51-like gene was detected in the genomes of all of the isolates, it was not expressed. Instead, TEM and OXA-23-like were the major β-lactamases responsible for MEM resistance. Overall, 37 isolates expressed TEM and OXA-23-like at the same time, while half of the remaining resistant strains produced only TEM and the other half expressed only OXA-23-like.

**TABLE 1:** Detection of β-lactamases in 50 clinical isolates

PCR positive (*n*, %)	RT-qPCR positive (*n*, %)	MIC of MEM (μg/mL)						
		Carbapenem-resistant				Carbapenem-sensitive		
		16	8	4		0.25	0.125	0.06
TEM, OXA-23-like, OXA-51-like (37, 74%)	TEM, OXA-23-like (37, 74%)	5	14	18		0	0	0
TEM, OXA-51-like (4, 8%)	TEM (4, 8%)	0	1	3		0	0	0
OXA-23-like, OXA-51-like (4, 8%)	OXA-23-like (4, 8%)	1	0	3		0	0	0
OXA-51-like (5, 10%)	None (5, 10%)	0	0	0		1	3	1

Although most of the isolates harbored these two resistance genes concurrently, the expression of *yiaD* still decreased under the pressure of MEM (*P < *0.001). Nonetheless, the extent of the reduction was less in resistant strains than in sensitive strains (Fig. 2B). Moreover, a correlation was found between the MIC value and the change in the expression level of *yiaD* in bacteria stimulated by MEM. Specifically, lower MIC values (from 0.06 μg/mL to 0.25 μg/mL) resulted in a more obvious reduction in the transcription of *yiaD* and vice versa (Fig. 2C).

### Loss of YiaD decreased the susceptibility of AB5116 to MEM.

To investigate whether YiaD helps A. baumannii adapt to the stress of MEM, its coding gene was knocked out from the genome of the carbapenem-susceptible strain AB5116. The MIC of AB5116 to MEM increased by 4-fold after *yiaD* knockout, from 0.125 μg/mL to 0.5 μg/mL (Table 2). Complementation of *yiaD* restored the susceptibility of this strain to MEM.

**TABLE 2:** MIC of strains used in this study to MEM

Strain	MIC (μg/mL)
AB5116	0.125
AB5116Δ*yiaD*::kan	0.5
AB5116Δ*yiaD*::kan+*yiaD*	0.125

### YiaD affected A. baumannii colony morphology.

**FIG 3:**
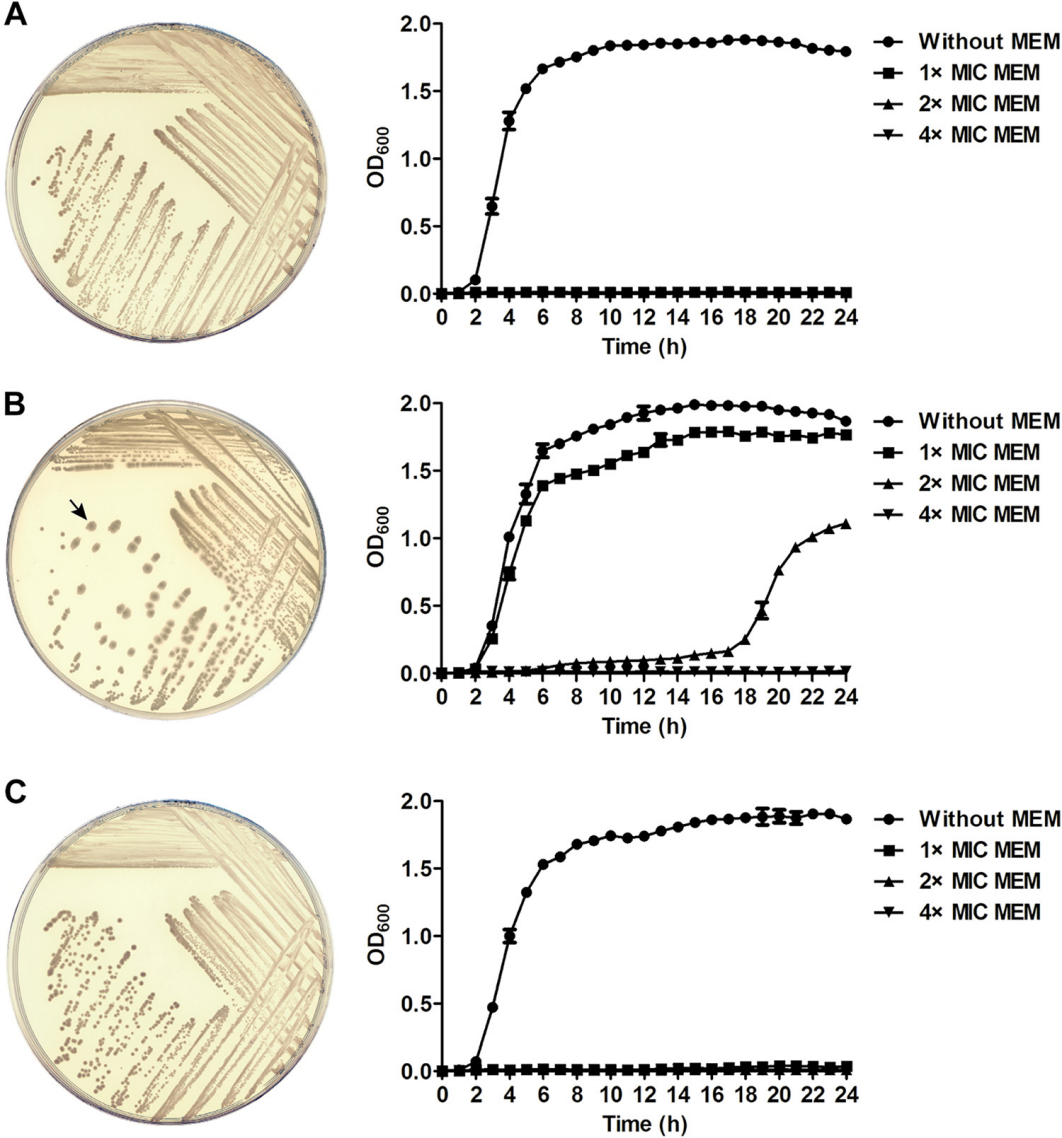
Growth of AB5116 (A), *yiaD* deletion mutant (B), and *yiaD* complementary bacteria (C) on agar plates and in LB broth. Bacteria were inoculated on LB agar plates and incubated overnight at 37°C. The arrow points to a typical irregular colony of the strain after loss of the *yiaD* gene. Growth curves were generated for bacteria cultivated in LB broth supplemented with 1 ×, 2 ×, and 4 × MIC of MEM, as well as without MEM at 37°C with shaking at 200 rpm. The OD_600_ was recorded every hour. The experiment was conducted in triplicate, and the results are presented as the means ± SD.

Loss of YiaD did not affect the growth performance of AB5116 in broth, as indicated by the average OD_600_ reaching 1.8 to 2.0 in the stationary phase (Fig. 3, without MEM). Surprisingly, the phenotype of colonies formed by the *yiaD* deletion mutant was changed. As shown in Fig. 3A and C, the colonies were round, regular, and medium-sized when YiaD was present but were enlarged and irregular with rims that were no longer compact after *yiaD* knockout (Fig. 3B).

When cultivated in LB broth, the three strains showed similar growth rate, regardless of whether the *yiaD* gene was expressed or not. Nevertheless, when MEM was present in the medium, bacterial growth was inhibited by MEM except AB5116Δ*yiaD*::kan. The reproduction speed of the *yiaD* deletion mutant under the stress of 0.125 μg/mL (1 × MIC) MEM was close to that in normal LB broth. The mutant strain could also survive at a concentration of 2 × MIC (0.25 μg/mL), but with a conspicuously delayed growth rate (OD_600_ 1.155 at 24 h). However, bacterial growth was prevented by 0.5 μg/mL (4 × MIC) MEM. For AB5116 and the *yiaD* complementary strain, no bacteria could grow in the presence of any concentration of MEM. These results were consistent with those of the MIC test.

### Loss of YiaD increased the survival rate of A. baumannii to MEM.

**FIG 4:**
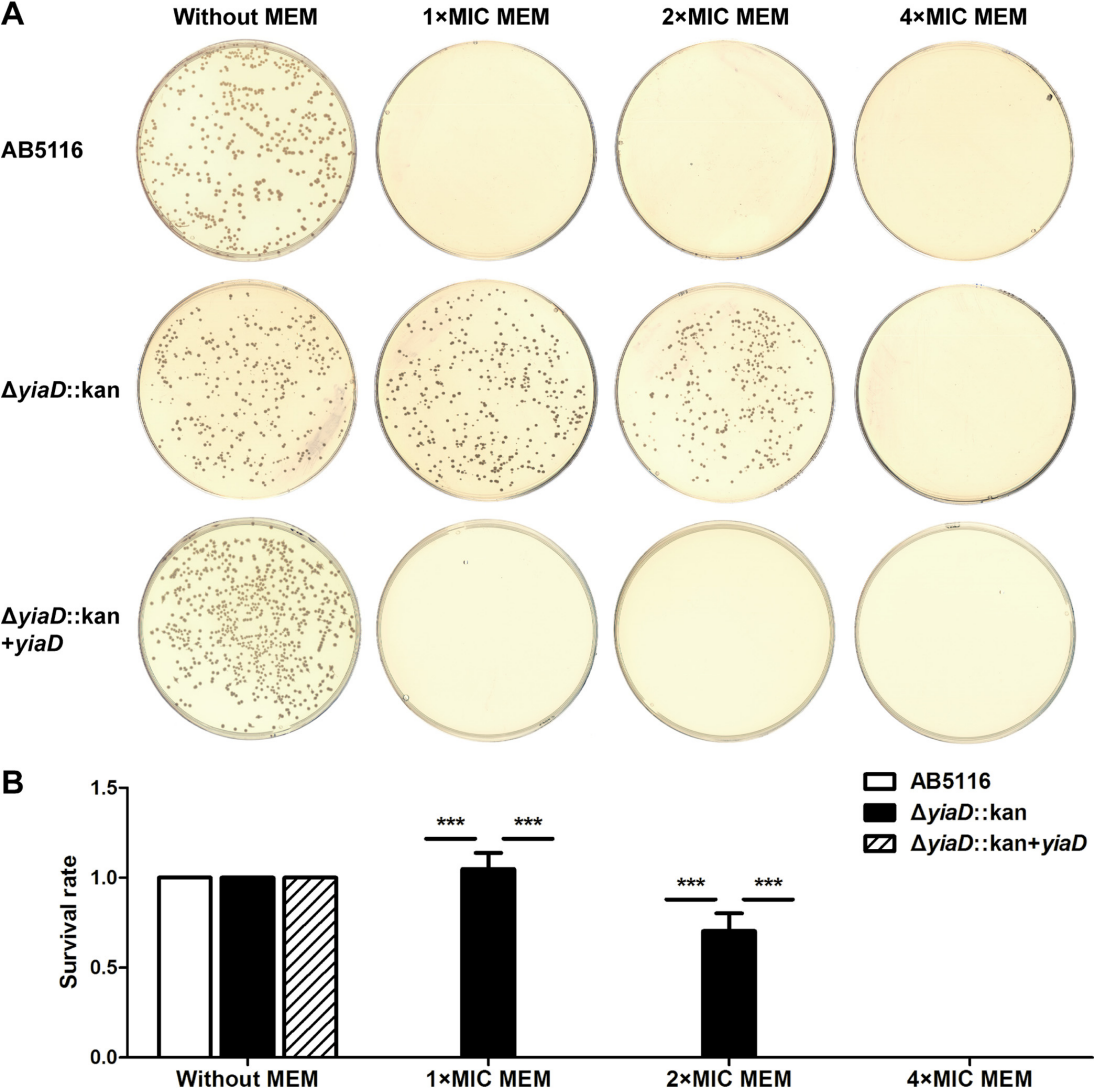
Survival rate assay of AB5116, *yiaD* deletion mutant, and complementary strain when cells were challenged with 1 ×, 2 ×, and 4 × MIC of MEM. (A) Overnight cultured bacterial cells were diluted to 10^−4^, and 100 μL of each dilution was spread on LB plates supplemented with different concentrations of MEM. Bacteria grown on plates without added antibiotics were used as controls. Plates were incubated overnight at 37°C, after which the results for the 10^−4^ dilution were recorded. (B) The number of colonies on each plate was counted. Survival rate was calculated by dividing the CFU/mL on the MEM plate by that on the control plate. The experiment was repeated in triplicate, and the results shown are the means ± SD. ***, *P < *0.001.

Survival rate assays were conducted to confirm whether the deletion of *yiaD* confers an advantage to bacterial survival in the presence of MEM. Antibiotic concentrations of 1 ×, 2 ×, and 4 × MIC were tested. After overnight culture on the 4 × MIC plates, none of the isolates formed colonies. The same result was observed for the wild-type and *yiaD* complementary strains grown on plates supplemented with 1 × and 2 × MIC of MEM (Fig. 4A). However, the survival rates for the *yiaD* deletion mutant reached 1.048 and 0.703 at these two concentrations, respectively, which was significantly higher than those of the *yiaD* expression groups (*P < *0.001) (Fig. 4B).

### YiaD contributed to biofilm formation.

**FIG 5:**
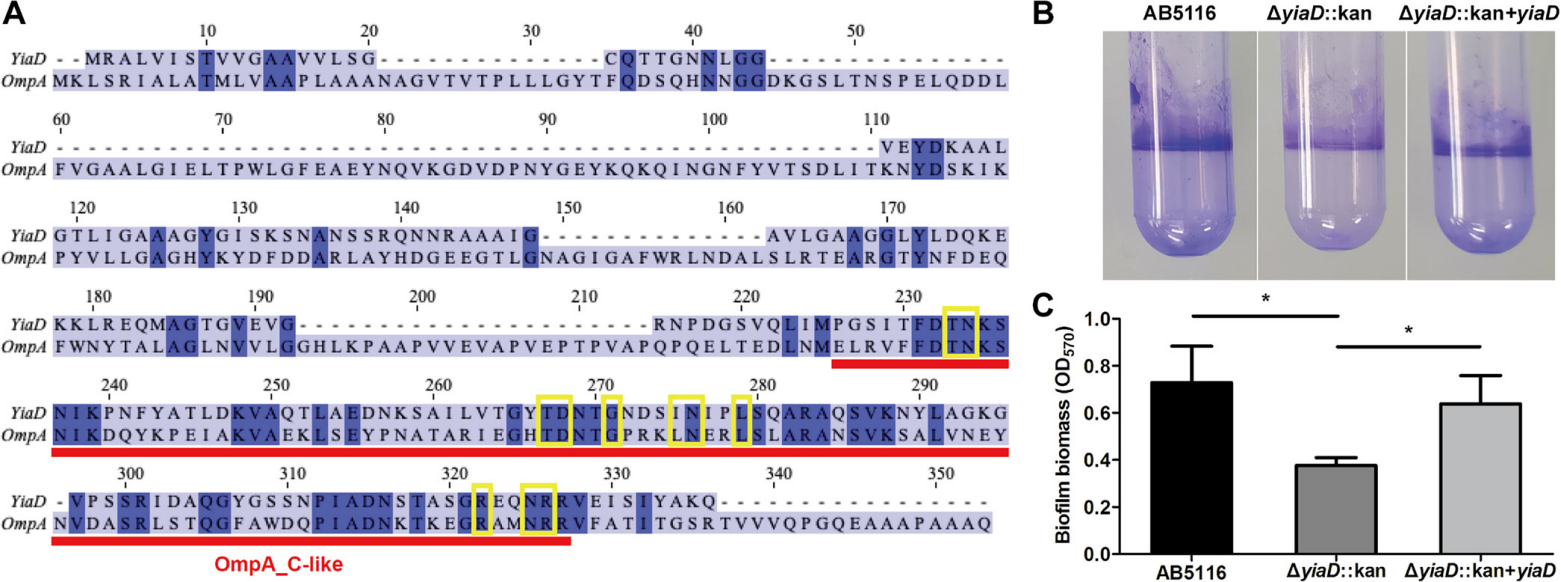
Sequence alignment of YiaD with OmpA and biofilm formation assay of AB5116 wild-type, Δ*yiaD*::kan, and Δ*yiaD*::kan + *yiaD* strains. (A) Protein sequences of OmpA and YiaD were compared using ClustalW. Sequences were analyzed based on information published in the NCBI protein database (https://www.ncbi.nlm.nih.gov/protein). The deep blue background denotes the same amino acids between two sequences. The red bar represents the OmpA_C-like structure, which has peptidoglycan binding domains similar to the C-terminal domain of the outer membrane protein OmpA. Yellow boxes indicate the ligand binding site (chemical binding) on OmpA. (B) Crystal violet staining of strains grown in plastic culture tubes at 37°C for 24 h. (C) Biofilm biomass measured by crystal violet staining at OD_570_. Asterisks denote significant differences in biofilm formation (*P < *0.05).

To identify more functions of YiaD in correlation with antibiotic resistance, the protein sequence was compared with those of other OMPs. The results revealed that YiaD is an OmpA-like protein with an OmpA-like carboxy-terminal (C-terminal) domain (Fig. 5A). In accordance with a previous report, the C-terminal domains of both OmpA and YiaD shared a conserved OmpA-like C-terminal domain structure (24). In addition, the chemical binding site showed high similarity with OmpA. These findings indicated that YiaD might exhibit semblable functions as OmpA.

Biofilm formation is an important virulence factor that is closely related to antibiotic resistance in A. baumannii (25). During biofilm production by this opportunistic pathogen, OmpA plays a key role in reversible attachment to surfaces (1). Because YiaD has a similar structure to OmpA, it was speculated that the former might also contribute to biofilm production by this bacteria. A biofilm formation assay revealed that *yiaD* deletion resulted in decreased biofilm production. Specifically, the biomass of biofilm decreased from an average of 0.729 at OD_570_ to 0.376 upon loss of *yiaD*. However, complementation of the mutant by ectopic expression of *yiaD* from a plasmid restored biofilm formation to the same level as that of the parental strain (Fig. 5B and C).

## DISCUSSION

Bacterial drug resistance has been a problem for decades, and various kinds of bacteria, including A. baumannii, have been found to evolve into MDR forms subsequent to antibiotic use (26). Moreover, many studies have reported XDR and PDR A. baumannii isolates (7, 27, 28). A. baumannii is a dangerous nosocomial pathogen that can survive in various environments. This organism is also naturally competent for DNA uptake and has high rates of natural transformation, which result in it easily becoming antibiotic resistant (26). Most studies on A. baumannii antibiotic resistance conducted to date have focused on the mechanisms of drug resistance based on identified resistant strains. Although sensitive bacteria require a long time to adapt to environments before showing the resistance phenotype, little is known about the adaptation process.

A previous study reported that a large number of independent mutations occurred through *in vivo* evolution in MDR Staphylococcus aureus strains isolated from an inpatient who had been treated with vancomycin (29). Similar results were also found for MDR and XDR Mycobacterium tuberculosis strains under drug pressures (30). These mutations might be compensatory changes, under the stress induced by antibiotics, and may involve the evolutionary process of resistance, though the mechanisms of these changes are not well understood (26). Moreover, what happens within the process when bacteria are exposed to antibiotic stress, as well as which factors promote the mutations, is unknown.

Acquisition of resistance to carbapenems, which are among the antimicrobials of last resort for treatment of multidrug infections, is increasing among A. baumannii strains, compounding the problem of nosocomial infections caused by this pathogen (7). Multiple intrinsic and acquired carbapenem resistance mechanisms have been revealed in A. baumannii, such as production of various carbapenemases, decreased permeability, overexpression of efflux pumps, and alterations of antibiotic target sites (7, 18, 31). In this study, we focused on the evolutionary process that lead to the likelihood of A. baumannii obtaining antibiotic resistance. To accomplish this, the carbapenem-sensitive strain AB5116 was treated with sub-MIC levels of MEM and then subjected to RNA-Seq analysis.

Infiltration into bacterial cells is the first step in antibiotics exerting an antimicrobial effect. As a Gram-negative bacterium, A. baumannii is surrounded by an outer membrane that acts as a permeability barrier against many substances, including antibiotics. OMPs are key routes through which antibiotics enter the outer membrane (17, 21), and no other antibiotic resistance mechanisms were found in the investigated strain. Thus, the changes in OMPs were investigated after treatment with MEM. Among the 12 investigated OMPs, only three coding genes, *carO*, *yiaD*, and *oprD* showed significant changes in expression. CarO is an 8-stranded beta barrel-shaped channel protein in the outer membrane that mediates the influx of β-lactams into A. baumannii (32). Mutations in CarO or its reduced expression have been shown to be related to carbapenem-resistant A. baumannii (32–35). OprD is a protein orthologous to a porin in Pseudomonas aeruginosa that can transport amino acids and imipenem to bacterial cells (36). OprD was first identified in CRAB isolates (37) and is now frequently reported in MDR and PDR clinical strains (38, 39). Conversely, little is known about YiaD. Hence, the main focus of this study was to identify the function of YiaD and the influences of its decreased expression.

In Escherichia coli, YiaD has been identified as a putative peptidoglycan-associated lipoprotein that is a multicopy suppressor of the *bamD* gene (40). YiaD is not essential for cell growth (40), but loss of this gene results in reduced swarming ability in E. coli (41). Further, its function in antibiotic resistance is unknown. YiaD was previously reported to exhibit the densest edges in the protein interaction network, along with OmpA and β-lactamase OXA-23, in A. baumannii (24). Similarly, our RNA-Seq data analysis revealed that *yiaD* had a high fragments per kilobase of transcript per million mapped reads (FPKM) value in the absence of MEM, with an average of 9,501.09, indicating that it encoded an OMP protein that was abundant in A. baumannii. *yiaD* was remarkably reduced under the stress of sub-MIC of MEM. Moreover, when the MEM treatment time was extended to 8 h, the expression level of *yiaD* decreased significantly with prolonged antibiotic stimulation time. We speculated that YiaD might have a similar function as CarO, which functions as an entry channel to transport MEM into A. baumannii cells. To protect themselves, strains of A. baumannii inhibit the influx of MEM by decreasing the expression of *yiaD*. MEM showed time-dependent antimicrobial activity, and prolonged infusion of this antibiotic was reportedly associated with higher clinical improvement rates and lower mortality (42). The continuous reduced expression of *yiaD* may contribute to the adaptation of A. baumannii to harsh environments containing MEM.

Furthermore, the reductive expression of *yiaD* under the pressure of MEM was not only observed in carbapenem-sensitive strains but also in more clinical CRAB isolates that produced two β-lactamases, TEM and OXA-23. These enzymes have strong carbapenem-hydrolyzing activity and can spread via horizontal transfer through plasmid or chromosomal carriage, resulting in resistance globally (43, 44). Despite producing TEM and OXA-23, all of the clinical CRAB strains showed prominent decreased expression of *yiaD* in the presence of sub-MIC of MEM. This finding demonstrated that YiaD might have a continuous protective response to MEM stimulation from an early stage. However, the decreased degree of *yiaD* transcription in the five sensitive clinical strains was similar to that in AB5116 and much lower than that in resistant isolates. In addition, as the MIC value increased, *yiaD* showed less decreased expression in clinical isolates. We speculated that TEM and OXA-23 executed the carbapenem-hydrolyzing function; however, they might not be able to destroy the antibiotic in a short time, which would result in a certain concentration of MEM existing along with the bacteria. For self-protection, A. baumannii strains could downregulate the expression of *yiaD* to inhibit the penetration of MEM into bacterial cells. Under such conditions, high-level resistance could be achieved via a synergistic effect of reduced uptake of antibiotics due to changed porin expression and the production of these degrading enzymes (45).

After inactivation of *yiaD* in AB5116, the MIC of MEM increased. Similar results were previously observed in the carbapenem-resistant strain AB5075 (24). Both the growth curve test and the survival rate assay confirmed the increase in MIC when *yiaD* was missing from the genome of A. baumannii. These findings revealed that reduced expression of *yiaD* resulted in a low-level of MEM resistance in A. baumannii. Although the MIC did not reach an obvious resistance level when YiaD was lost, it did promote bacteria to overcome MEM stress; therefore, it might be involved in the development of adaptive resistance in A. baumannii in coordination with other factors such as CarO. These findings highlighted the importance of YiaD in adaptive resistance of MEM in A. baumannii and further suggested that this protein helped bacteria to overcome the challenge of MEM stress.

In addition to the increased MIC, changes in the morphology of the bacterial colonies were also observed. Specifically, colonies of the Δ*yiaD* mutant were enlarged and had irregular rims. These changes could be associated with the function of YiaD, which was present in high abundance in the outer membrane. YiaD has been predicted as an OmpA-like protein that shares a conserved C-terminal domain structure with OmpA (24). Specifically, the chemical binding site in the C-terminal domain of YiaD had high similarity with that of OmpA (Fig. 5A). This conserved OmpA-like C-terminal domain has been shown to be related to the peptidoglycan layer in bacterial cells and is important for the maintenance of bacterial surface integrity (1, 40, 46). Thus, it could be involved in the maintenance of colony shape.

Notably, although YiaD and OmpA had similar structures at the C-terminal domain, their functions in the MEM resistant phenotype were not the same. OmpA has been reported to be involved in the resistance phenotype of A. baumannii by allowing slow diffusion of β-lactam antibiotics (47), and its loss causes strains to be more susceptible to carbapenems (21, 48). However, low-level resistance to MEM was observed in YiaD inactivation strains of A. baumannii. These findings suggest that even though a conserved C-terminal domain is shared by YiaD and OmpA, the effects of MEM stimulation and the mechanism of response differ between these two proteins, possibly because of other parts of the protein structure.

Biofilm formation is another dominant characteristic in A. baumannii with various functions, such as interacting with host cells, promoting infections, enhancing tolerance to extracellular stresses, and increasing antibiotic resistance (49–51). OmpA is a prominent porin involved in biofilm formation (52). As an OmpA-like protein, we were curious about the role that YiaD plays in biofilm production in A. baumannii. As expected, the level of biofilm formation decreased when strains lost *yiaD*, indicating that it participates in the process of biofilm formation by this pathogen.

In conclusion, the OmpA-like protein YiaD was found to respond to the stimulus of MEM in this study. Moreover, its expression continued to decrease with prolonged stimulation time, and decreased transcription was observed in both carbapenem sensitive and resistant strains, with the latter producing the β-lactamases TEM and OXA-23. Lack of *yiaD* also contributed to low-level resistance in A. baumannii, and thus, *yiaD* may be involved in adaptive resistance. Lack of *yiaD* was also correlated with the morphology of bacterial colonies and biofilm formation in this pathogen. Therefore, YiaD is an important OMP that may participate in various routes that lead to MEM resistance evolution in A. baumannii.

This study mainly focused on the role of YiaD with respect to MEM adaptive resistance. Its contribution to resistance in other carbapenem drugs, as well as its correlation with CarO and OprD, will be further investigated in future studies.

## MATERIALS AND METHODS

### Bacterial strains and culture conditions.

AB5116 is a clinical carbapenem-susceptible A. baumannii strain isolated from the First Affiliated Hospital of Xi’an Jiaotong University. This strain has the typical phenotype (Gram-negative coccobacillus, aerobic, oxidase-negative, catalase-positive, nonfermenting, forming grayish, smooth, and mucoid colonies at 37°C). This organism has a MIC of IPM and MEM of 0.125 μg/mL and was previously reported as strain S2 (53). Another 50 A. baumannii clinical isolates were collected from Shaanxi Provincial People’s Hospital and used to verify changes in *yiaD* expression level under the pressure of MEM. Escherichia coli DH5α was obtained from TIAGEN Biotech Co. Ltd. (Beijing, China). Luria-Bertani (LB, Oxoid, UK) medium was used for routine bacterial culture, and Mueller-Hinton (MH, Oxoid) broth medium was used MIC testing. When necessary, kanamycin and carbenicillin were added to the LB medium at final concentrations of 50 μg/mL and 100 μg/mL, respectively. The bacteria were cultured at 37°C and stored at −80°C in LB broth with 20% glycerol.

### Determination of MIC for MEM.

The antibiotic susceptibility of clinical strains was determined using the Vitek 2 system (bioMérieux, Marcyl’étoile, France), and the broth macrodilution method described by the Clinical and Laboratory Standards Institute (CLSI) was used to confirm the MIC values of MEM for all strains used in this study (54). Bacterial suspensions were incubated at 37°C with shaking at 200 rpm for 24 h.

### Identification of β-lactamase genes in clinical strains.

β-lactamase genes in 50 clinical strains were identified as previously described (53), with some modifications. Briefly, the genomic DNA of all of the clinical isolates was extracted using a TIANamp Bacteria DNA Kit (TIANGEN, Beijing, China) according to the manufacturer’s instructions. The presence of 14 β-lactamases genes was then detected by PCR. Specifically, the samples were analyzed for the presence of five class A genes (*bla*_KPC_, *bla*_GES_, *bla*_TEM_, *bla*_SHV_, *bla*_CTX_), four class B genes (*bla*_IMP_, *bla*_VIM_, *bla*_SIM-1_, *bla*_NDM_), and five class D genes (*bla*_OXA-23-like_, *bla*_OXA-24-like_, *bla*_OXA-48-like_, *bla*_OXA-51-like_, *bla*_OXA-58-like_) using the primers listed in Table 3. PCR was performed using 2 × *Taq* Master Mix (Novoprotein, Suzhou, China) in a total volume of 20 μL containing 10 μL of 2 × *Taq* Master Mix, 0.5 μM each primer, and 2 μL of DNA template. The amplification program consisted of predenaturation at 94°C for 2 min, followed by 30 cycles of denaturation at 95°C for 20 s, annealing at the appropriate temperature for 20 s, extension at 72°C for 1 min, and final elongation at 72°C for 5 min.

**TABLE 3:** Primers used in this study

Primer	Sequence (5′ to 3′)	Application	Reference
yiaDqPCR-F	TGGCTGAAGATAACAAGAGCGC	RT-qPCR for *yiaD*	This study
yiaDqPCR-R	CGATACGGCTAGATGGAACACC		
16S rRNA-F	CAGCTCGTGTCGTGAGATGT	RT-qPCR for 16S rRNA	This study
16S rRNA-R	CGTAAGGGCCATGATGACTT		
yiaDup-F	GTGATGAGTGCACACCTTAAAGTG	Construction of Δ*yiaD*::kan	This study
yiaDup-R	TCCAGCCTACACAATCATTGCATGCCTCCT		
yiaDdw-F	GGAGGATATTCATATGGACCTAAGATCTAGTT	Construction of Δ*yiaD*::kan	This study
yiaDdw-R	GGCAGATGACATCGTGTATTACC		
kanFRT-F	ATTGTGTAGGCTGGAGCTGCTTC	Construction of Δ*yiaD*::kan	This study
kanFRT-R	GGTCCATATGAATATCCTCCTTAGTTCC		
yiaDscreen-F	GGCCCCGAATATCCAAAATCC	Confirmation of Δ*yiaD*::kan	This study
yiaDscreen-R	CCAGCAAGCACCAATAAAGGG		
yiaDcomp-F	GGTACCGTTGACATATAAATGGCCCC	Complementation of *yiaD*	This study
yiaDcomp-R	GTCGACAAAACCACCCGAAGGT		
*bla*_KPC_-F	TGTCACTGTATCGCCGTC	Detection of *bla*_KPC_	(59)
*bla*_KPC_-R	CTCAGTGCTCTACAGAAAACC		
*bla*_GES_-F	GTTTTTGCAATGTGCTCAACG	Detection of *bla*_GES_	(60)
*bla*_GES_-R	TGCCATAGCAATAGGCGTAG		
*bla*_TEM_-F	ATAAAATTCTTGAAGACGAAA	Detection of *bla*_TEM_	(60)
*bla*_TEM_-R	GACAGTTAGCAATGCTTAATCA		
*bla*_SHV_-F	GCCTTTATCGGCCCTCACTCAAG	Detection of *bla*_SHV_	This study
*bla*_SHV_-R	TTAGCGTTGCCAGTGCTCGATCA		
*bla*_CTX_-F	CGTCACGCTGTTGTTAGGAA	Detection of *bla*_CTX_	(60)
*bla*_CTX_-R	ACCGTCGGTGACGATTTTAG		
*bla*_IMP_-F	GAAGGCGTTTATGTTCATAC	Detection of *bla*_IMP_	(61)
*bla*_IMP_-R	GTACGTTTCAAGAGTGATGC		
*bla*_VIM_-F	GTTTGGTCGCATATCGCAAC	Detection of *bla*_VIM_	(61)
*bla*_VIM_-R	AATGCGCAGCACCAGGATAG		
*bla*_SIM-1_-F	TACAAGGGATTCGGCATCG	Detection of *bla*_SIM-1_	(60)
*bla*_SIM-1_-R	TAATGGCCTGTTCCCATGTG		
*bla*_NDM_-F	GCAGCTTGTCGGCCATGCGGGC	Detection of *bla*_NDM_	(61)
*bla*_NDM_-R	GGTCGCGAAGCTGAGCACCGCAT		
*bla*_OXA-23-like_-F	GATGTGTCATAGTATTCGTCG	Detection of *bla*_OXA-23-like_	(60)
*bla*_OXA-23-like_-R	TCACAACAACTAAAAGCACTG		
*bla*_OXA-24-like_-F	GGTTAGTTGGCCCCCTTAAA	Detection of *bla*_OXA-24-like_	(60)
*bla*_OXA-24-like_-R	AGTTGAGCGAAAGGGGATT		
*bla*_OXA-48-like_-F	GCGTGGTTAAGGATGAACAC	Detection of *bla*_OXA-48-like_	(61)
*bla*_OXA-48-like_-R	CATCAAGTTCAACCCAACCG		
*bla*_OXA-51-like_-F	ATGAACATTAAAGCACTC	Detection of *bla*_OXA-51-like_	(60)
*bla*_OXA-51-like_-R	CTATAAAATACCTAATTGTTC		
*bla*_OXA-58-like_-F	AAGTATTGGGGCTTGTGCTG	Detection of *bla*_OXA-58-like_	(60)
*bla*_OXA-58-like_-R	CCCCTCTGCGCTCTACATAC		
TEMqPCR-F	TTCCGGCTGGCTGGTTTATT	RT- qPCR for TEM	This study
TEMqPCR-R	TGACTCCCCGTCGTGTAGAT		
OXA-23-likeqPCR-F	TAATGCTCTAAGCCGCGCAA	RT-qPCR for OXA-23-like	This study
OXA-23-likeqPCR-R	TTCTCCAATCCGATCAGGGC		
OXA-51-likeqPCR-F	TCGGCCTTGAGCACCATAAG	RT-qPCR for OXA-51-like	This study
OXA-51-likeqPCR-R	GCCATAACCAACACGCTTCA		

### RNA extraction.

A fresh overnight culture of AB5116 was 1:100 diluted in LB broth and then grown to an optical density (OD) at 600 nm (OD_600_) of 0.4, after which 0.5 × MIC of MEM was added into the bacterial suspension. For RNA-Seq and preliminary verification of *yiaD* expression, bacteria were treated with MEM for 30 min. For further investigation of changes in of *yiaD* expression over time, bacterial cells were divided into five groups and exposed to MEM for 0.5 h, 1 h, 2 h, 4 h, or 8 h. To confirm the *yiaD* expression level in clinical isolates, bacteria were stimulated with MEM for 2 h. Control samples were subjected to the same conditions without MEM. RNA was extracted using RNAprotect Bacteria Reagent (Qiagen, Hilden, Germany) and an RNeasy minikit (Qiagen) according to the manufacturer’s protocols, after which it was subjected to DNase I (Thermo Scientific, Vilnius, Lithuania) treatment to ensure there was no DNA present.

### RNA sequencing analysis.

Bacterial mRNAs from three replicates were enriched by removing ribosome RNA (rRNA) using a Ribo-Zero Magnetic Kit (Epicentre, San Diego, CA, USA). First-strand cDNA was obtained from purified mRNA using an iScript cDNA synthesis kit (Bio-Rad, Hercules, CA, USA) with random primers, followed by synthesis of second-strand cDNA using DNA polymerase I, RNase H, dNTP and buffer (TaKaRa, Beijing, China). Next, the cDNA fragments were sequenced using an Illumina HiSeq 2500 by Gene Denovo Biotechnology Co. (Guangzhou, China) after ligation to Illumina sequencing adapters.

High-quality clean reads were obtained from raw data by filtering, after which rRNA mapped reads were removed following alignment with the reference genome of AB5116 (accession number CP091173) using Bowtie 2 (55). The remaining clean reads were then aligned to the AB5116 genome sequence using TopHat2 (56). Gene expression levels were normalized by FPKM, and differences in gene expression were then calculated based on these levels. DEGs were selected according to a fold change ≥ 2 and a false discovery rate (FDR) < 0.05 between the two groups (challenged with and without MEM). Data were further subjected to enrichment analysis of GO functions (http://www.geneontology.org) and KEGG pathways (http://www.genome.jp/kegg). The raw RNA-Seq data were submitted to the SRA database under the BioProject number PRJNA797559.

### RT-qPCR.

RT-qPCR was performed to verify the expression levels of the *yiaD* gene under the stress of MEM. cDNAs were acquired from DNA-free total RNAs by reverse transcription using a RevertAid First Strand cDNA Synthesis Kit (Thermo Scientific) with a random hexamer primer. The gene expression level was then standardized relative to the transcription level of 16S rRNA using the primers listed in Table 3. SYBR Select Master Mix (Thermo Scientific) was used in a final volume of 20 μL, and qPCR was conducted using an Agilent Mx3005P QPCR System (Agilent Technologies, Santa Clara, CA, USA) under the following conditions: initial incubation at 50°C for 2 min and at 95°C for 2 min, followed by 40 cycles of 95°C for 15 s, 55°C for 15 s, and 72°C for 1 min. Relative abundance was determined by the ΔΔCt method using the MEM-treated values in comparison with non-treated values in each strain. RT-qPCR was also conducted to investigate the expression of positive β-lactamase genes in 50 clinical strains as mentioned above. All assays were carried out in triplicate.

### Construction of AB5116Δ*yiaD*::kan mutant.

The *yiaD* gene was knocked out from the AB5116 genome by allelic replacement using the Rec_Ab_ system as described by Tucker et al. (57), with brief modifications. Briefly, the upstream and downstream homology arms flanking the coding sequence of *yiaD* were amplified from AB5116 by PCR using PrimeSTAR Max DNA polymerase (TaKaRa) instead of by synthesis. The Kanamycin-resistance (Km^R^) selection marker was then amplified from pKD4, after which overlapping PCR was conducted to generate the deletion cassette of the upstream-Km^R^-downstream fragment. The deletion cassette was subsequently electroporated into pAT02-containing competent AB5116 cells, and bacteria were grown in LB medium supplemented with 2 mM IPTG. Mutants were selected on LB agar plates with kanamycin and subsequently confirmed by PCR using 2 × *Taq* Master Mix (Novoprotein, Shanghai, China). The *yiaD* deletion was verified by Sanger sequencing.

### Complementation of *yiaD*.

The *yiaD* gene of AB5116 was amplified by PCR using PrimeSTAR Max DNA polymerase. The fragment was cloned into pAT03, an isopropyl β-D-1-thiogalactopyranoside (IPTG)-induced expression vector in A. baumannii (57), using DNA Ligation Kit Ver.2.1 (TaKaRa), and then further transformed into DH5α to obtain the plasmid pAT03-*yiaD*. The competent AB5116Δ*yiaD*::kan cells were transformed with the target plasmid, which was confirmed by sequencing, and subsequently grown on LB agar containing carbenicillin and IPTG. Complementation of *yiaD* was confirmed by PCR using the yiaDqPCR primer set.

### Growth curve.

Growth curves of the strains were determined to investigate the effects of the *yiaD* gene on bacterial growth. Briefly, overnight cultures of AB5116, AB5116Δ*yiaD*, and the *yiaD* complemented strain were calibrated to a concentration of 5 × 10^5^ CFU/mL in fresh LB broth, after which they were cultured at 37°C while shaking at 200 rpm. During culture, the OD_600_ was measured every hour from 0 h to 24 h using an UNICO UV-2100 spectrophotometer (UNICO, Shanghai, China). The inhibitory role of MEM was confirmed by adding 1 × MIC, 2 × MIC, or 4 × MIC of this antibiotic into the bacterial suspensions.

### Survival rate analysis.

A survival rate assay was conducted as described by Yu et al. (58), with slight modification. Briefly, the target bacterial cells from fresh overnight LB cultures were diluted to 10^−4^, after which 100 μL of each dilution was spread on LB plates containing 1 × MIC, 2 × MIC, and 4 × MIC of MEM, or no antibiotics. Plates were then incubated overnight at 37°C, after which the colonies were enumerated. The survival rate was calculated by dividing the CFU/mL on the MEM plate by the CFU/mL on the control plate.

### Biofilm formation analysis.

The contribution of *yiaD* to biofilm formation in A. baumannii was tested by a biofilm production assay as previously described (50), with some modifications. Briefly, 2 mL of bacterial suspensions were cultured for 24 h in plastic culture tubes at 37°C. Biofilms were then stained with 0.1% crystal violet after washing with phosphate buffer saline (PBS). The results were recorded as the mean ± SD of the OD at 570 nm after triplicate repeats.

### Statistical analysis.

Results were statistically analyzed using GraphPad Prism 8.0 (San Diego, CA, USA). The relative expression level of *yiaD* was reported as the mean ± SD of the data from different independent experiments. Quantitative analysis of *yiaD* expression in AB5116 was conducted using one-way ANOVA, while that in clinical strains was analyzed by *t*-tests. The survival rates of different strains under different MEM concentrations were compared using two-way ANOVA. The difference in the biofilm biomass formed by the strains was analyzed by *t*-tests. A *P* value < 0.05 was considered statistically significant for all tests.

### Data availability.

The genome sequence of AB5116 was submitted to GenBank with the accession number CP091173. Additionally, the RNA-Seq raw data were submitted to the SRA database with the BioProject number PRJNA797559.
